# Random survival forest predicts survival in patients with metastatic laryngeal and hypopharyngeal cancer and the prognostic benefits of surgery and radiotherapy

**DOI:** 10.7150/jca.103793

**Published:** 2025-01-01

**Authors:** Yusheng Wang, Chaofan Li, Feilun Yang, Minjie Gong, Jingkun Qu, Ruiping Ma, Zhenzhen Hu, Miao Lou, Xiaoyong Ren, Guoxi Zheng, Yanxia Bai, Ya Zhang, Jin Hou

**Affiliations:** 1 Department of Otolaryngology Head and Neck Surgery, The Second Affiliated Hospital of Xi'an Jiaotong University, Xi'an 710004, China.; 2 The Comprehensive Breast Care Center, The Second Affiliated Hospital of Xi'an Jiaotong University, Xi'an 710004, China.; 3 Department of Otolaryngology Head and Neck Surgery, The First Affiliated Hospital of Xi'an Jiaotong University, Xi'an 710061, China.; 4 Department of Otorhinolaryngology Head and Neck Surgery, Shaanxi Provincial People's Hospital, China.

**Keywords:** Laryngeal and Hypopharyngeal Cancer, Distant Metastases, SEER, Primary Surgery, Machine Learning

## Abstract

**Background:** Laryngeal and hypopharyngeal cancers are prominent within head and neck malignancies. The diagnosis of distant metastasis (DM) invariably signals poor prognosis, underscoring the need to optimize current treatment approaches.

**Methods:** Patient data for metastatic laryngeal and hypopharyngeal cancer were extracted from the SEER database (2000-2020). Cox regression and propensity score matching (PSM) analyses identified independent prognostic factors and performed stratified survival analyses based on the receipt of primary tumor surgery and radiotherapy. A random survival forest (RSF) model was subsequently developed to predict patient survival.

**Results:** A total of 1,626 patients were included. PSM-based stratified analysis revealed that primary tumor surgery significantly improved survival in patients under 70 years and those with primary laryngeal cancer. Radiotherapy enhanced survival across all age groups, with a benefit primarily for patients with primary laryngeal cancer and squamous-cell carcinoma (SCC). The RSF model demonstrated robust predictive performance, highlighting chemotherapy, primary tumor surgery, and radiotherapy as the top three factors influencing patient survival.

**Conclusion:** The clinical and pathological features of metastatic laryngeal/hypopharyngeal cancer were systematically analyzed using an artificial intelligence (AI) model to predict survival. Subgroup analyses identified patients most likely to benefit from primary tumor surgery and radiotherapy. These findings may guide the development of personalized treatment strategies, potentially improving the prognosis of patients with DM.

## Introduction

Head and neck cancer (HNC) ranks as the sixth most common cancer globally 1, affecting multiple anatomical sites including the oral cavity, oropharynx, larynx, and hypopharynx. Among these, laryngeal and hypopharyngeal cancers are the second most prevalent respiratory malignancies, following lung cancer 2.

Although HNC is primarily localized, distant metastasis (DM) occurs infrequently, with an incidence ranging from 2.8% to 23.8%, most commonly affecting the lungs, bones, and liver 3, 4. The presence of DM is a critical prognostic indicator, often associated with a dismal outcome 3, 5. Patients with DM are rarely cured, with most receiving palliative treatments, and their median overall survival (OS) is approximately one year under first-line Cetuximab-based chemotherapy 6-8. According to the National Comprehensive Cancer Network (NCCN) guidelines for HNC (2020) 9, the standard first-line therapies for metastatic laryngeal and hypopharyngeal cancers include platinum-based chemotherapy combined with epidermal growth factor receptor (EGFR) inhibitors (e.g., cetuximab) 8, 10 or immune checkpoint inhibitors (e.g., pembrolizumab) 11. For oligometastatic disease, locoregional treatments, including surgery, radiation therapy, or ablative therapies, are recommended. Uncontrolled locoregional tumor progression in this anatomical region, which governs vital functions such as breathing, swallowing, speaking, and cranial nerve function, can lead to significant morbidity. This progression often results in severe complications like dyspnea and dysphagia, contributing to a marked deterioration in quality of life and, in many cases, premature mortality 12. This highlights the vital role of primary tumor surgery. While the survival benefit of palliative primary tumor resection has been established for several malignancies 13, 14, its efficacy for laryngeal and hypopharyngeal cancers remains unclear. Similarly, while radiotherapy is an established treatment for metastatic disease 15, its role and the specific subgroups of patients who benefit most from it have not been fully explored. Despite the generally poor prognosis, patients with metastatic laryngeal and hypopharyngeal cancer should not be considered immediately fatal. A proactive, combined treatment approach is essential to prolong survival and preserve key laryngeal functions. Given the rarity of metastatic laryngeal/pharyngeal cancer, its management has not been extensively investigated in randomized clinical trials (RCTs). Therefore, this study seeks to clarify the potential benefits of primary tumor surgery and radiotherapy for patients with DM.

Previous research, particularly RCTs, has predominantly focused on local-regional diseases, with limited studies addressing metastatic laryngeal/hypopharyngeal cancer. A few nomograms have been developed to estimate survival in patients with DM 16, though the accuracy of these models has not been thoroughly assessed, highlighting the need for more robust predictive tools. In populations where randomization is not feasible, it becomes critical to quantify the specific treatment effects, as observed survival benefits may be confounded by group-related factors rather than reflecting the true impact of the treatment. With the rise of computer science and artificial intelligence (AI) 17-19, novel prognostic models have emerged. Machine learning, in particular, has established itself as a powerful tool for survival prediction across various cancer types 20-23, with random survival forest (RSF)—a model based on decision trees—emerging as one of the most promising approaches. The RSF model is particularly suited for prognostic prediction across a wide range of diseases, leveraging internal data cross-validation to ensure prediction accuracy and prevent overfitting 24, 25. Additionally, it prioritizes the most influential prognostic factors, aiding in the identification of key determinants and enhancing clinical decision-making 24.

This study conducted an extensive analysis of the Surveillance, Epidemiology, and End Results (SEER) database, focusing on the long-term OS and disease-specific survival (DSS) of patients with metastatic laryngeal/hypopharyngeal cancer. It further explored the impact of various prognostic factors on survival outcomes. To minimize confounding variables, propensity score matching (PSM) was employed, allowing for stratified survival analyses based on the presence or absence of primary tumor surgery and radiotherapy. An RSF model was then developed to predict survival in this patient cohort. This research marks a significant advancement in the development of AI-based clinical models, aiming to optimize long-term survival outcomes for patients with metastatic laryngeal/hypopharyngeal cancer while providing valuable insights into their prognosis.

## Materials and Methods

### Data source and study design

**Figure 1:**
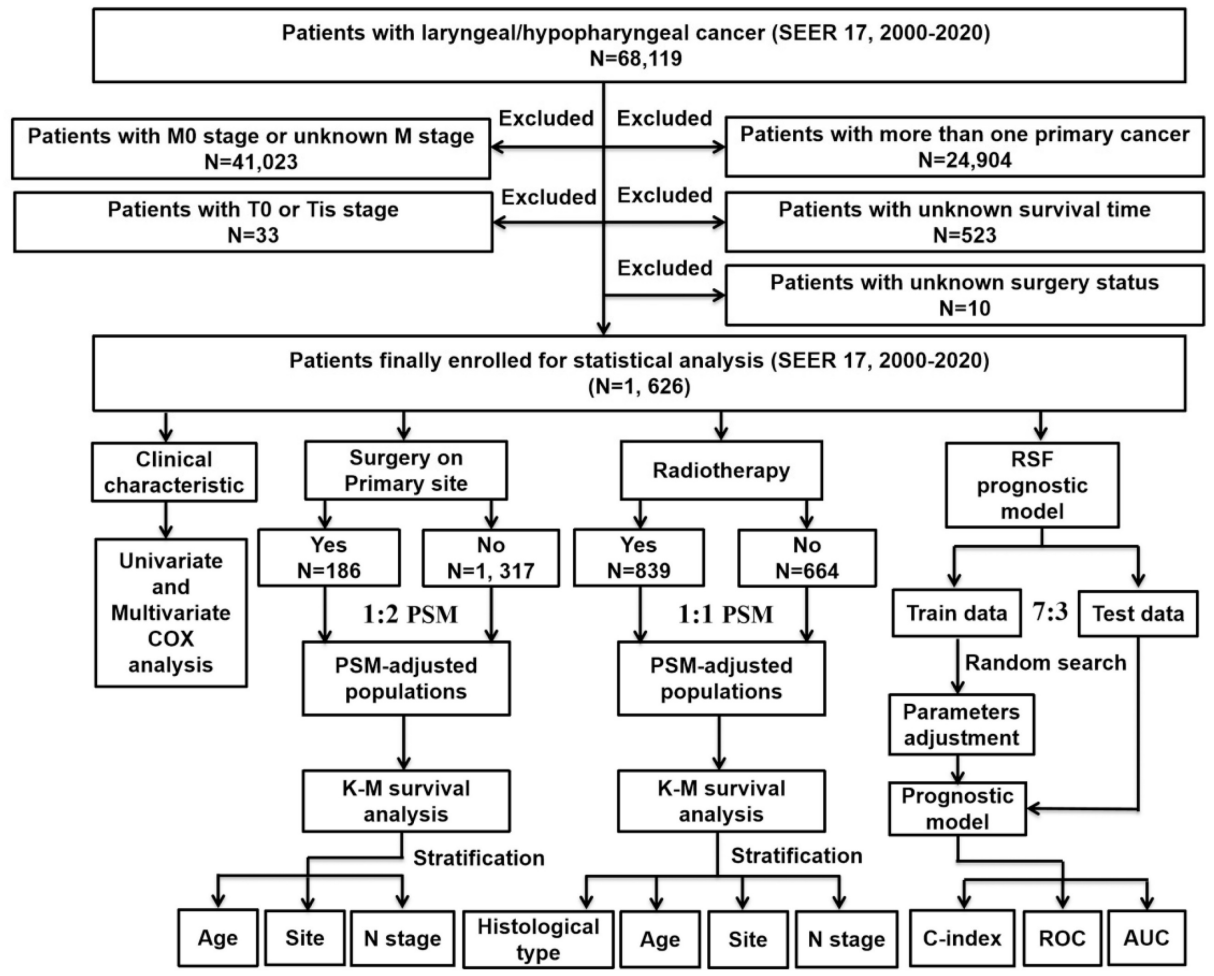
The flowchart of the study process and statistical analysis. SEER: the Surveillance Epidemiology and End Results; K-M: Kaplan-Meier; RSF: random survival forest; PSM: propensity score matching; C-index: concordance index; ROC: re**cei**ver operator characteristic curve; AUC: area under the curve.

The research design and analysis are outlined in the workflow (Figure 1). Data on patients with metastatic laryngeal or hypopharyngeal cancer were obtained from the SEER database (SEER 17 Regs study data, changes 2000-2020; version 8.4.1). Inclusion criteria were: 1) diagnosis of laryngeal or hypopharyngeal cancer; 2) histopathological and morphological evidence consistent with the International Classification of Cancer Diseases Edition III (ICD-O-3). Exclusion criteria included: 1) patients with M0 stage or unknown M stage; 2) patients with multiple primary tumors; 3) absence of essential clinical data such as survival months or surgery status; 4) patients with T0 stage or in situ cancer (Tis). Follow-up continued until the patient's death, loss to follow-up, or December 31, 2020.

### Statistical analysis

Univariate Cox regression models were used to explore the relationship between patient survival and various demographic and clinicopathological factors. Variables with significant differences (*P* < 0.05) were subsequently included in multivariate Cox analysis to assess hazard ratios (HR) and identify independent prognostic factors. All statistical analyses were performed using R programming language (version 4.0.2). Kaplan-Meier (K-M) survival curves were evaluated with the log-rank test, with two-tailed *P* < 0.05 considered statistically significant.

### Propensity score matching (PSM)

To reduce bias from unbalanced baseline characteristics and further evaluate the impact of primary tumor surgery and radiotherapy on patient prognosis, PSM was applied. This method is particularly useful for comparing unequally sized groups 26. Based on variables identified as significant in univariate Cox analysis, patients who underwent primary tumor surgery were matched with those who did not on a 1:2 ratio, and patients who received radiotherapy were matched with those who did not on a 1:1 ratio. The following parameters were used for matching: method = “nearest”, distance = “logit”, replace = FALSE, caliper = 0.05. Kaplan-Meier survival analysis and log-rank tests were then performed on the PSM-adjusted population.

### RSF model

The RSF model was developed using the least absolute shrinkage and selection operator (LASSO) regression analysis. The random forest-based predictor was constructed with the randomForestSRC package, and the out-of-bag (OOB) error was used as the model's performance metric. Patients were randomly divided into training and test sets in a 7:3 ratio. Model training was performed on the training set, and prediction accuracy was assessed on both the training and test sets. In the RSF algorithm, for each tree, a subset of candidate variables was randomly selected. Trees were grown until the size of the final node met a minimum number of events with distinct survival times. At each node, random candidate variables were chosen, and the set that maximized the log-rank statistics was used to split the branches. A random search strategy was employed for model training and parameter optimization to ensure model stability and to identify the most critical hyperparameters. Additionally, variable importance was ranked based on the calculation of the OOB error rate. To evaluate the performance of the RSF model, Harrell's concordance index (C-index) was calculated using OOB data 27. The C-index measures the model's ability to predict the timing of patient death, making it a key metric for survival prediction. The C-index ranges from 0.5 to 1, with a C-index of 1 indicating perfect concordance.

## Results

### Clinical characteristics of patients with metastatic laryngeal/hypopharyngeal cancer

A total of 1,626 eligible patients with metastatic laryngeal or hypopharyngeal cancer were included, comprising 1,331 men (81.86%) and 1,132 patients aged < 70 years (69.62%). Detailed demographic and clinicopathological characteristics are presented in Supplementary Table 1. Squamous cell carcinoma (SCC) was the dominant histological type, found in 90.96% of patients, with other histological types accounting for only 9.04%. The primary tumor sites were categorized as laryngeal (including supraglottis 38.13%, glottis 12.12%, subglottis 1.91%, and other laryngeal sites 15.99%) or hypopharyngeal (31.86%). Regarding tumor grade, approximately one-third of patients had grade II (moderately differentiated, 31.98%) or grade III/IV (poorly differentiated, 30.44%) tumors. The distribution of T stages was as follows: T1 8.30%, T2 19.99%, T3 20.60%, and T4 36.84%, while N stages were distributed as N0 14.95%, N1 16.91%, N2 48.03%, and N3 9.90%. Treatment modalities included chemotherapy in 939 patients (57.75%), radiotherapy in 852 (52.40%), primary tumor surgery in 189 (11.62%), regional lymph node surgery in 368 (22.63%), and distant site surgery (on metastatic tumors) in 69 (4.24%). Lung was the most common site of distant metastasis (622 cases, 38.25%), followed by bone (14.88%), liver (10.46%), distant lymph nodes (7.87%), other distant organs (4.06%), and brain (1.11%).

### Univariate and multivariate cox regression analyses

Univariate Cox regression analysis identified variables significantly associated with survival in patients with metastatic laryngeal/hypopharyngeal cancer, including age at diagnosis, histological type, marital status, N stage, primary site, tumor grade, treatment modalities, and metastasis sites (bone, liver, lung, brain) (Table 1).

Multivariate Cox regression analysis was then performed to account for confounding factors and identify independent prognostic factors for OS and DSS (Table 2). Single marital status, advanced N stage, and high pathological grade were significantly associated with worse OS. Additionally, primary tumors located in the supraglottis and other laryngeal sites, as well as metastasis to bone and liver, were linked to worse DSS. In terms of treatment, chemotherapy (OS: HR = 0.333, 95% confidence interval [CI] = 0.271-0.410, *P* < 0.001; DSS: HR = 0.477, 95% CI = 0.384-0.592, *P* < 0.001), radiotherapy (OS: HR = 0.512, 95% CI = 0.424-0.620, *P* < 0.001; DSS: HR = 0.668, 95% CI = 0.545-0.819, *P* < 0.001), and primary tumor surgery (OS: HR = 0.507, 95% CI = 0.366-0.703, *P* < 0.001; DSS: HR = 0.674, 95% CI = 0.494-0.921, *P* < 0.05) were all found to significantly improve both OS and DSS in patients with metastatic laryngeal/hypopharyngeal cancer.

### Benefits of primary tumor surgery in metastatic laryngeal/hypopharyngeal cancer

To evaluate the impact of primary tumor surgery on patients with DM, it is crucial to identify which patient subgroups may derive the greatest benefit from this intervention. Based on the Cox regression results, a further stratified analysis was conducted to assess prognosis differences and identify factors influencing surgical decision-making. A 1:2 PSM analysis was performed to correct for imbalances in baseline characteristics. Following PSM adjustment, the *P*-values for all covariates exceeded 0.05, indicating that baseline characteristics were well-matched (Supplementary Table 2).

**Figure 2:**
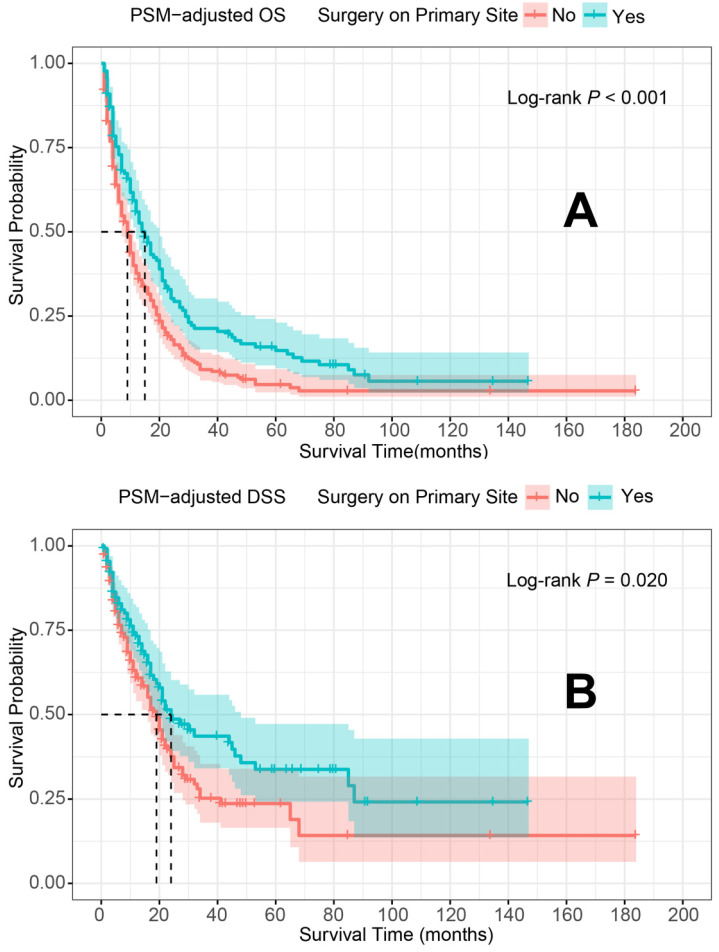
PSM-adjusted OS and DSS of the patients with and without primary tumor surgery. Kaplan-Meier survival analysis: A. OS of the patients; B. DSS of the patients. PSM: propensity score matching; OS: overall survival; DSS: disease-specific survival.

**Figure 3:**
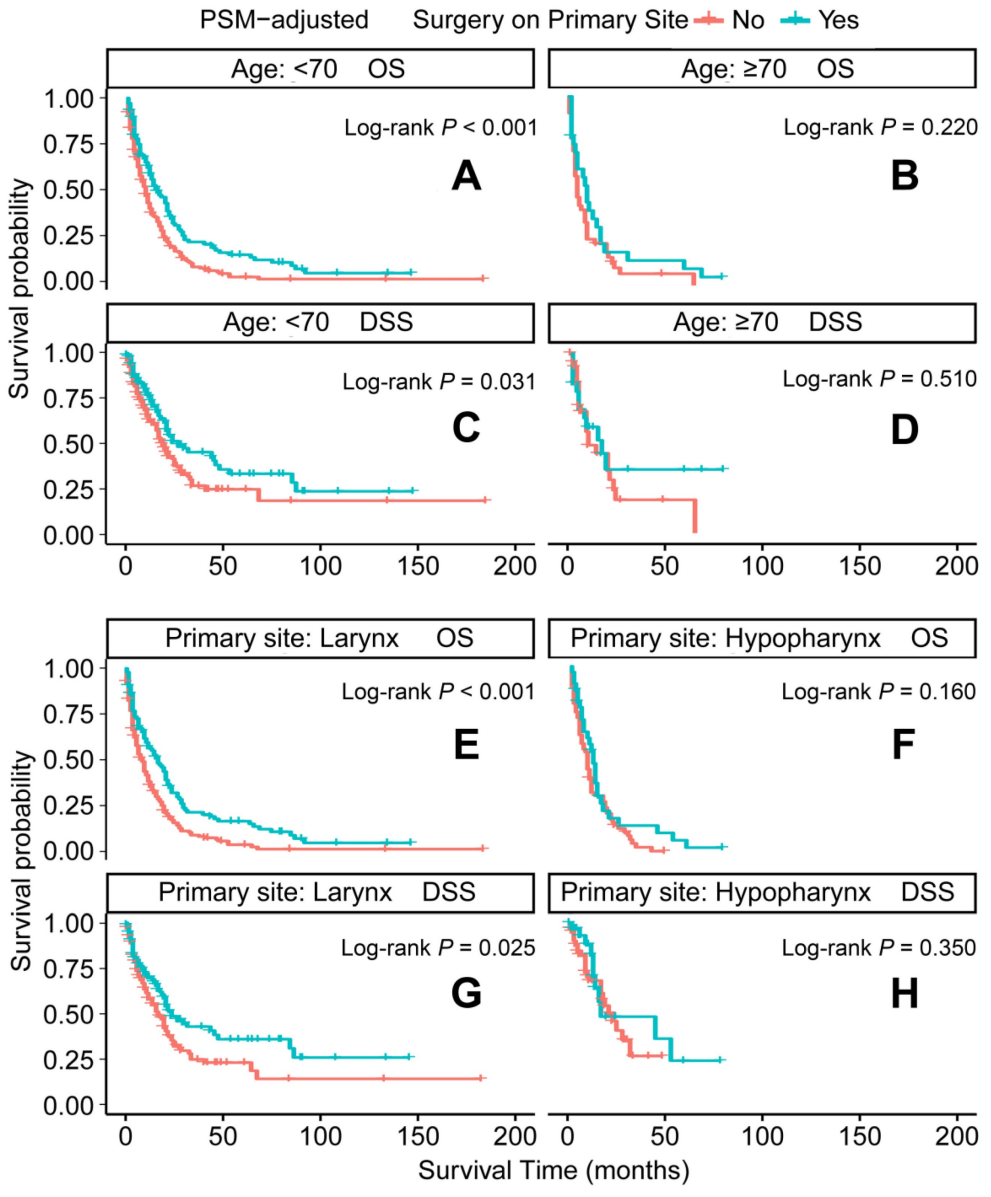
PSM-adjusted OS and DSS of the patients receiving primary tumor surgery (Stratified by age at diagnosis and primary site). Kaplan-Meier survival analysis: A. OS of the patients aged <70 years; B. OS of the patients aged ≥ 70 years; C. DSS of the patients aged <70 years, D. DSS of the patients aged ≥ 70 years; E. OS of the patients with primary laryngeal cancer; F. OS of the patients with primary hypopharyngeal cancer; G. DSS of the patients with primary laryngeal cancer, H. DSS of the patients with primary hypopharyngeal cancer. PSM: propensity score matching; OS: overall survival; DSS: disease-specific survival.

**Figure 4:**
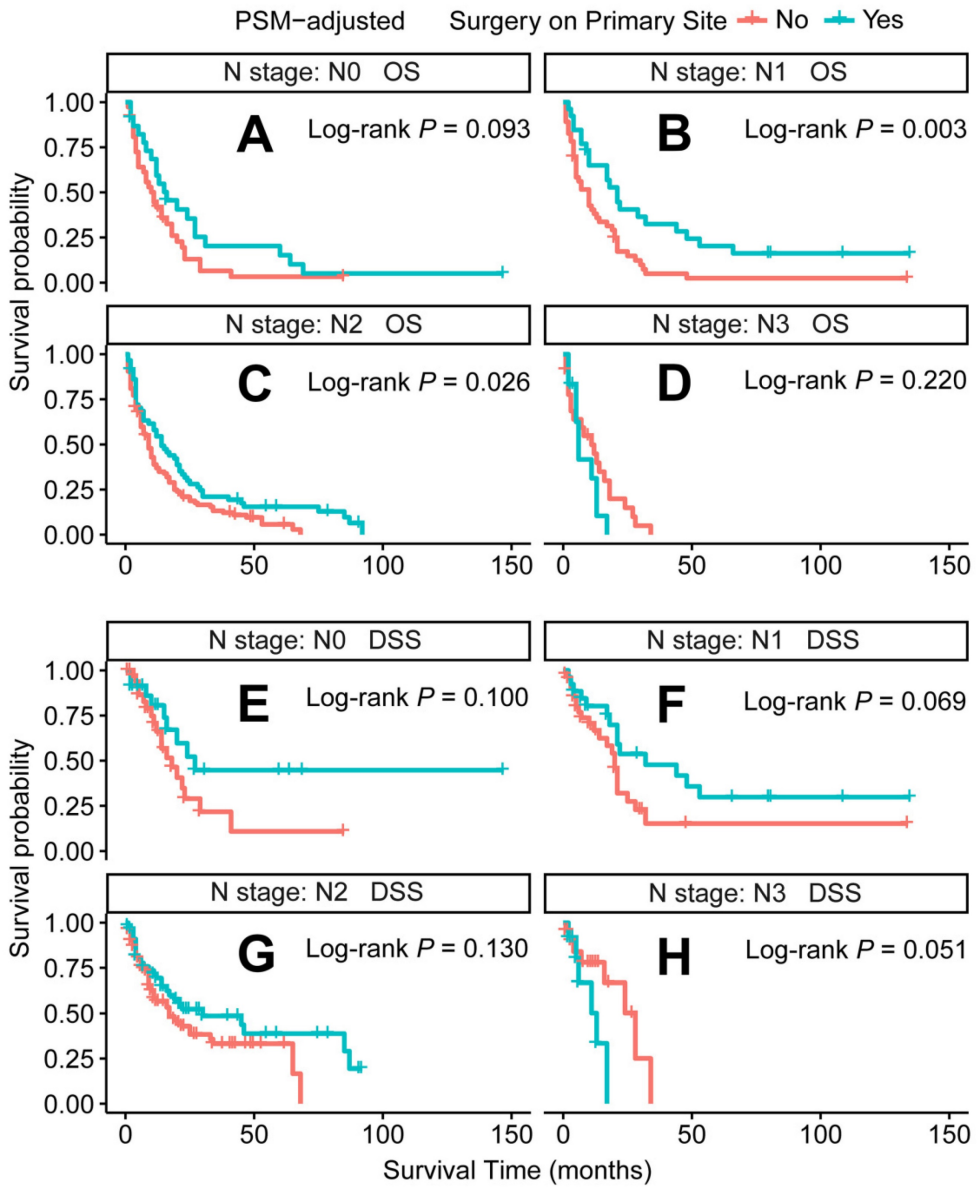
PSM-adjusted OS and DSS of the patients receiving primary tumor surgery (Stratified by N stage). Kaplan-Meier survival analysis: A. OS of the patients with N0 stage; B. OS of the patients with N1 stage; C. OS of the patients with N2 stage; D. OS of the patients with N3 stage; E. DSS of the patients with N0 stage; F. DSS of the patients with N1 stage; G. DSS of the patients with N2 stage; H. DSS of the patients with N3 stage. PSM: propensity score matching; OS: overall survival; DSS: disease-specific survival.

In the PSM-adjusted cohort, primary tumor surgery was associated with significant improvements in both OS and DSS for patients with metastatic laryngeal/hypopharyngeal cancer (Figure 2A and 2B). However, the survival benefits of primary tumor surgery varied across subgroups. Stratified Kaplan-Meier survival analysis revealed that for patients aged < 70 years, primary tumor surgery significantly improved both OS and DSS (Figure 3A and 3C), while no such benefit was observed for patients aged 70 years or older (Figure 3B and 3D). Regarding the primary tumor site, primary tumor surgery enhanced both OS and DSS for patients with laryngeal cancer (Figure 3E and 3G), but did not confer survival benefits for those with hypopharyngeal cancer (Figure 3F and 3H). Additionally, OS was significantly improved in patients with N1 and N2 stage disease who underwent primary tumor surgery (Figure 4B and 4C). The median OS for each subgroup, stratified by primary tumor surgery status, is presented in Supplementary Table 3.

### Benefits of radiotherapy in metastatic laryngeal/hypopharyngeal cancer

In the treatment of patients with laryngeal/hypopharyngeal cancer who developed DM, palliative chemotherapy is virtually universal 9, while radiotherapy is not as widely applied. To assess the specific benefits of radiotherapy, a detailed stratified matched analysis was conducted to correct for baseline imbalances between patients who received radiotherapy and those who did not. After performing 1:1 PSM, baseline characteristics were balanced, with *P*-values greater than 0.05 for all covariates, indicating uniformity between the groups (Supplementary Table 4).

**Figure 5:**
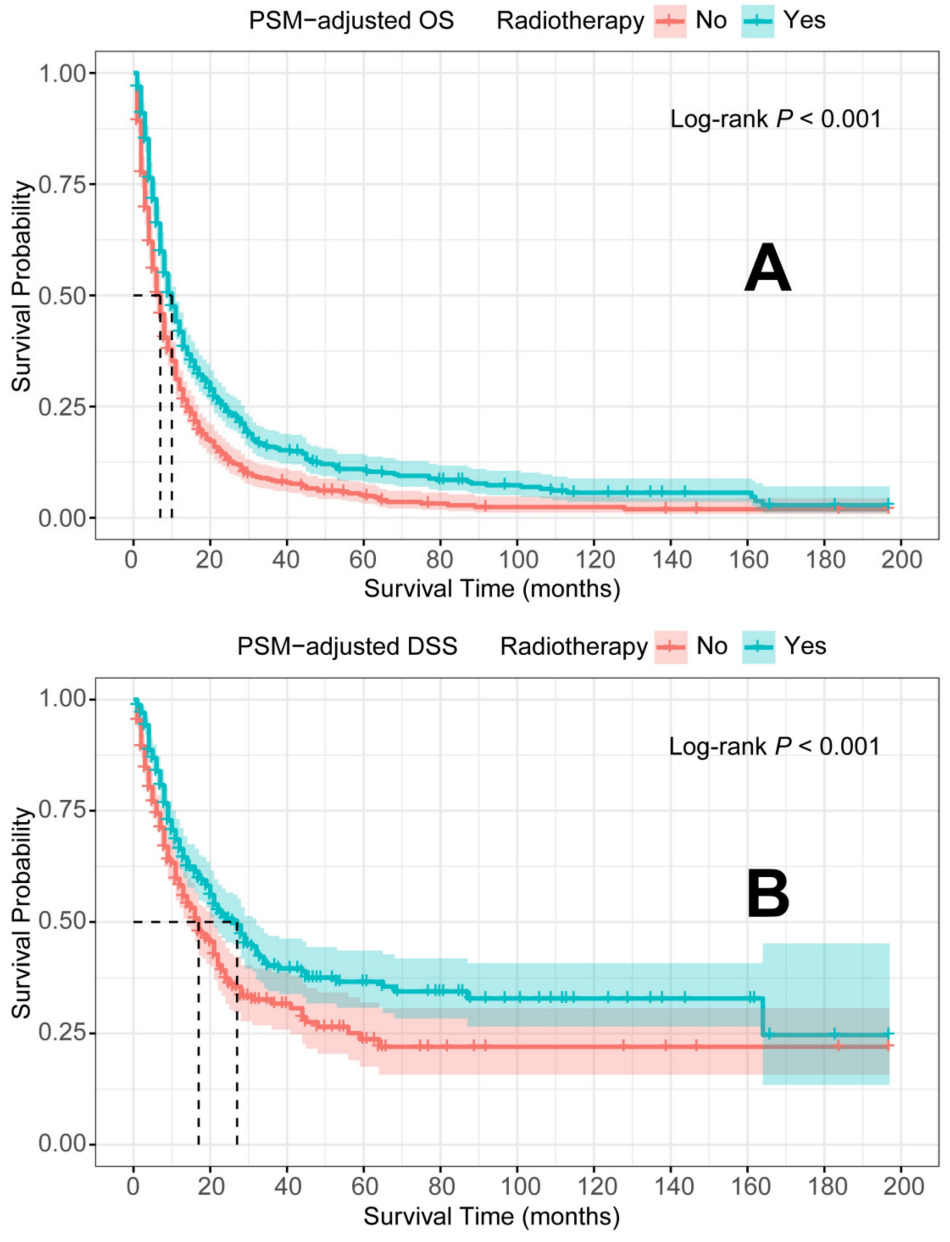
PSM-adjusted OS and DSS of the patients with and without radiotherapy. Kaplan-Meier survival analysis: A. OS of the patients; B. DSS of the patients. PSM: propensity score matching; OS: overall survival; DSS: disease-specific survival.

**Figure 6:**
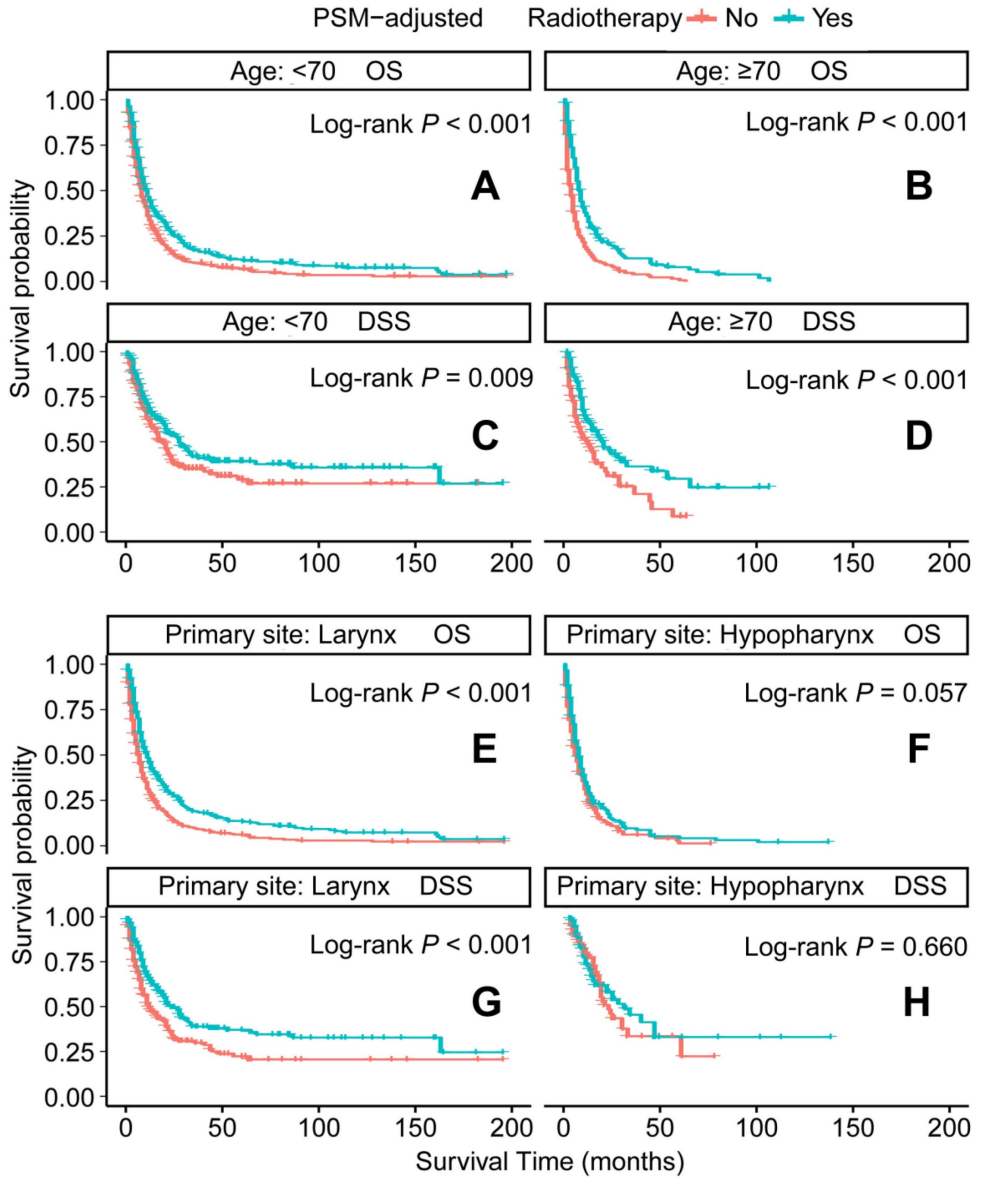
PSM-adjusted OS and DSS of the patients receiving radiotherapy (Stratified by age at diagnosis and primary site). Kaplan-Meier survival analysis: A. OS of the patients aged <70 years; B. OS of the patients aged ≥ 70 years; C. DSS of the patients aged <70 years, D. DSS of the patients aged ≥ 70 years; E. OS of the patients with primary laryngeal cancer; F. OS of the patients with primary hypopharyngeal cancer; G. DSS of the patients with primary laryngeal cancer, H. DSS of the patients with primary hypopharyngeal cancer. PSM: propensity score matching; OS: overall survival; DSS: disease-specific survival.

**Figure 7:**
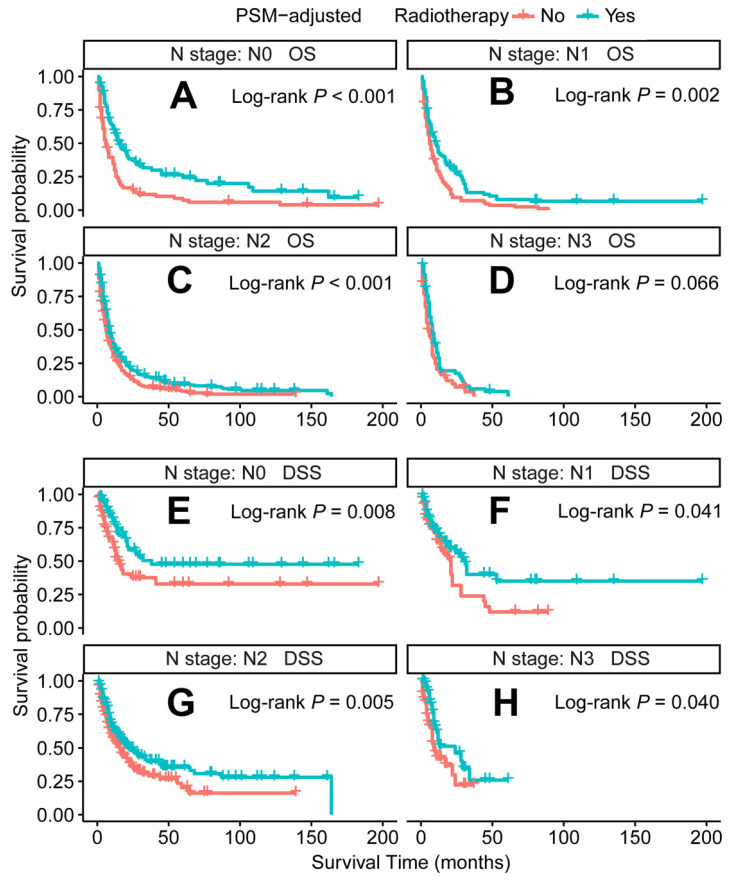
PSM-adjusted OS and DSS of the patients receiving radiotherapy (Stratified by N stage). Kaplan-Meier survival analysis: A. OS of the patients with N0 stage; B. OS of the patients with N1 stage; C. OS of the patients with N2 stage; D. OS of the patients with N3 stage; E. DSS of the patients with N0 stage; F. DSS of the patients with N1 stage; G. DSS of the patients with N2 stage; H. DSS of the patients with N3 stage. PSM: propensity score matching; OS: overall survival; DSS: disease-specific survival.

**Figure 8:**
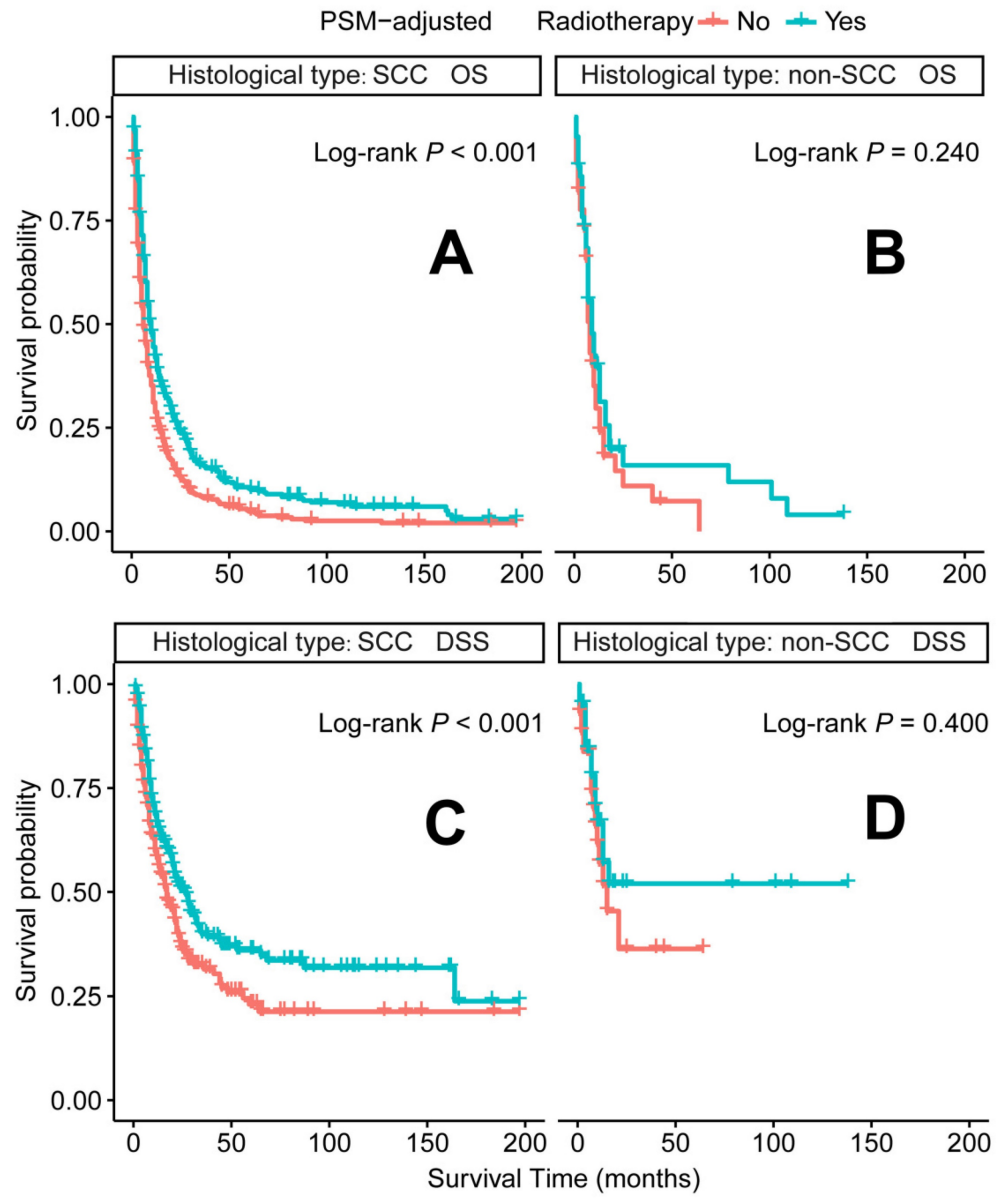
PSM-adjusted OS and DSS of the patients receiving radiotherapy (Stratified by histological type). Kaplan-Meier survival analysis: A. OS of the patients with SCC; B. OS of the patients with non-SCC; C. DSS of the patients with SCC; D. DSS of the patients with non-SCC. PSM: propensity score matching; OS: overall survival; DSS: disease-specific survival; SCC: squamous-cell carcinoma; non-SCC: all the histological types other than SCC.

In the PSM-adjusted cohort, radiotherapy was found to significantly improve both OS and DSS in patients with metastatic laryngeal/hypopharyngeal cancer (Figure 5A-B). Stratified K-M survival analysis showed that for patients of all age groups, radiotherapy provided significant improvements in OS (Figure 6A and 6B) and DSS (Figure 6C and 6D). For different primary tumor sites, radiotherapy improved both OS and DSS in patients with laryngeal cancer (Figure 6E and 6G), but did not offer benefits for those with hypopharyngeal cancer (Figure 6F and 6H). Furthermore, radiotherapy improved OS in patients with N0 to N2 stages of disease (Figure 7A-C) and improved DSS in patients across all N stages (Figure 7E-H). Regarding histological types, radiotherapy improved OS and DSS only in patients with SCC (Figure 8A and 8C), while non-SCC patients did not derive significant benefits (Figure 8B and 8D). Specific median OS (in months) for each subgroup of patients, based on radiotherapy status, are provided in Supplementary Table 5.

### RSF

**Figure 9:**
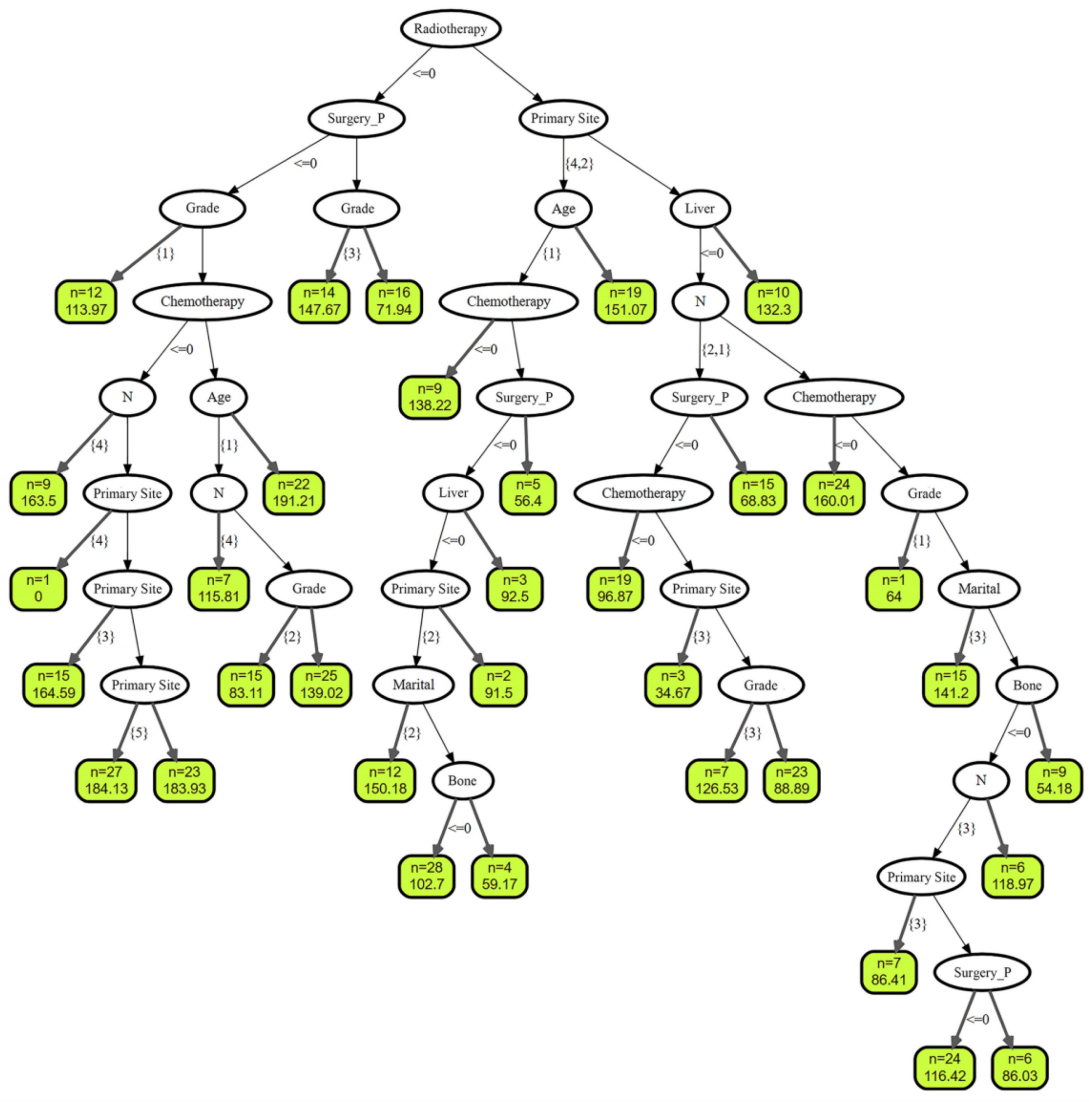
Graphical presentation of the Random Survival Forest (RSF) algorithm.

**Figure 10:**
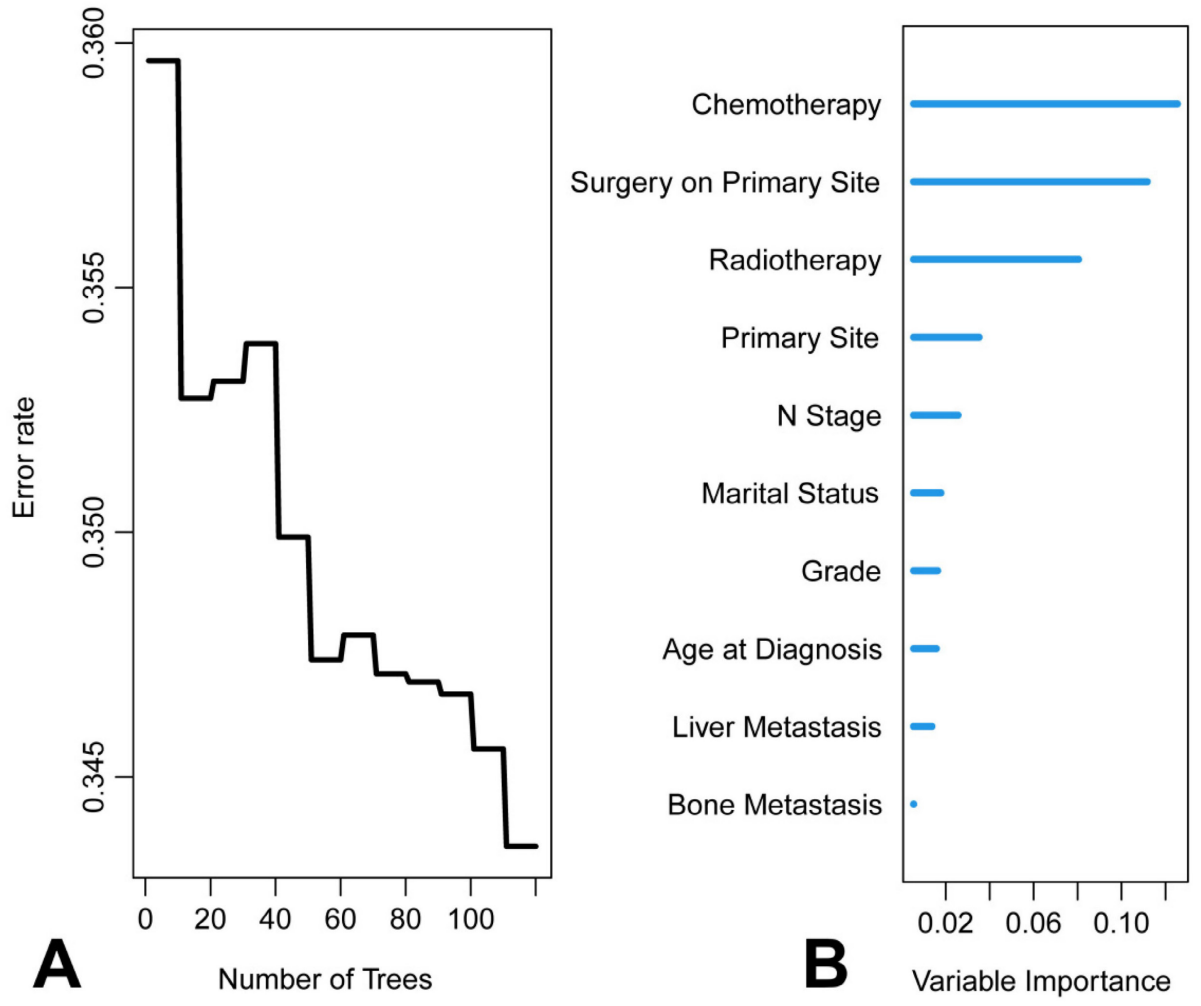
Results of random survival forests (RSF). A. Relationship between the prediction error rate for RSF and number of classification trees. B. The rank of features based on how they influence the survival of patients with metastatic laryngeal/hypopharyngeal cancer.

**Figure 11:**
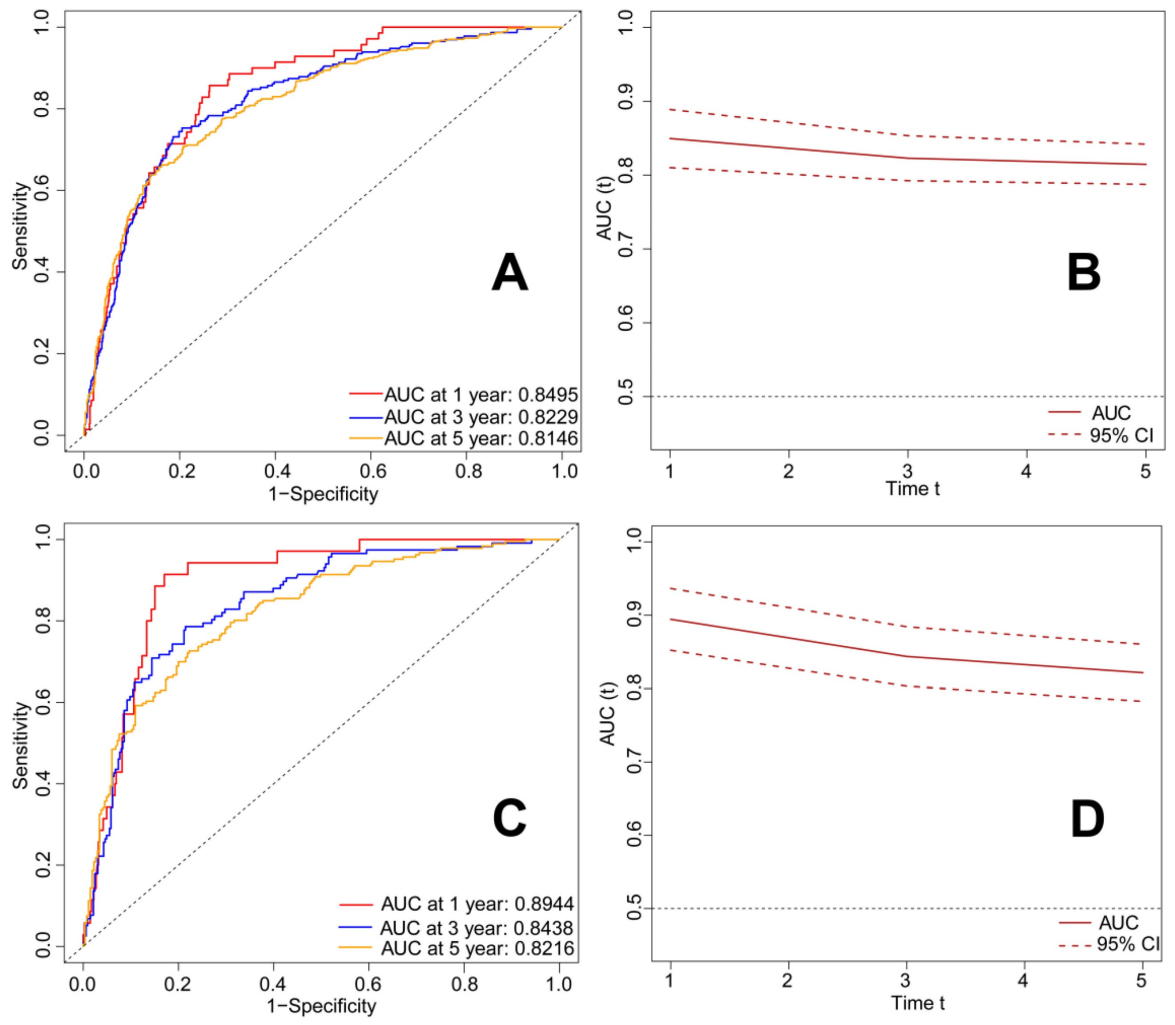
ROC curves and time-dependent AUC of patients with metastatic laryngeal and hypopharyngeal cancer in the train set and test set. A.ROC curve for predicting 1-, 3-, and 5-year OS rates in the patients with metastatic laryngeal and hypopharyngeal cancer in the train set; B. Time-dependent AUC in the train set; C. ROC curve for predicting 1-, 3-, and 5-year OS rates in the patients with metastatic laryngeal and hypopharyngeal cancer in the test set; D. Time-dependent AUC in the test set. OS: overall survival; ROC: receiver operator characteristic curve; AUC: area under the curve.

**Figure 12:**
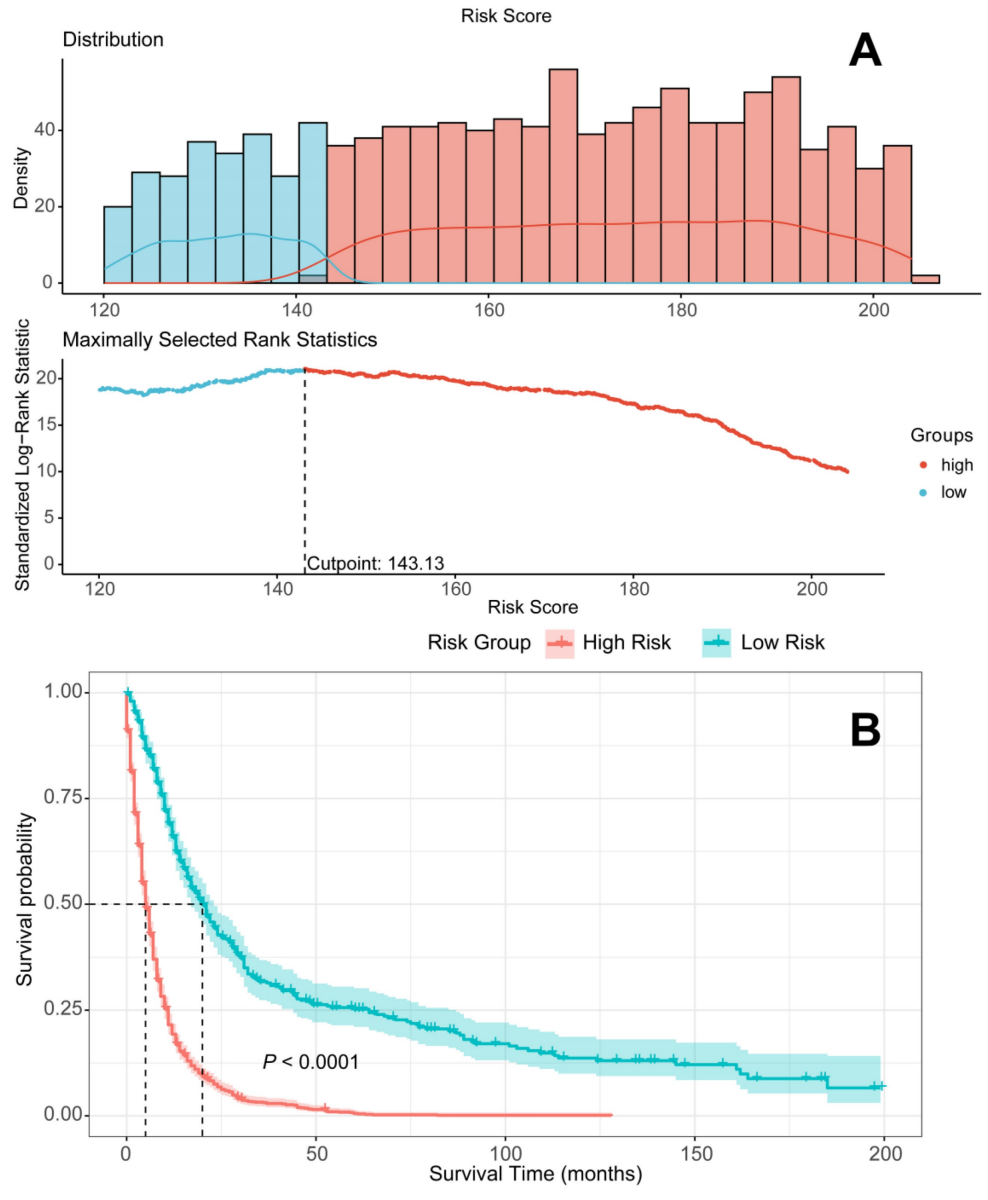
Patients devided into high-risk and low-risk groups according to the Random Survival Forest model. A. Patients divided into high-risk and low-risk groups according to risk scores; B Survival analysis to compare the prognosis of the two groups.

The graphical presentation of the RSF model is visualized in Figure 9. Figure 10A shows the error rate of the RSF model as a function of the number of trees, while Figure 10B highlights the ten most important variables affecting the survival of patients with metastatic laryngeal/hypopharyngeal cancer. Chemotherapy, primary tumor surgery, and radiotherapy were identified as the top three predictors of survival, demonstrating the most significant impact on model predictions. Overall, the RSF model demonstrated strong performance in survival prediction, with the area under the time-dependent receiver operating characteristic curve (time-dependent AUC) exceeding 0.8 (Figure 11B and 11D). The AUC values at 1-, 3-, and 5-year for the training set were 0.8495, 0.8229, and 0.8146, respectively (Figure 11A), and for the test set, they were 0.8944, 0.8438, and 0.8216 (Figure 11C). Survival predictions were performed using our model, and patients were classified into high-risk and low-risk groups (Figure 12A). Patients in the low-risk group had significantly better prognoses than those in the high-risk group (*P* < 0.001), suggesting that our model is capable of accurately predicting patient mortality risk (Figure 12B).

## Discussion

Laryngeal and hypopharyngeal cancers are predominant subtypes of HNC, and when diagnosed at the metastatic stage, they typically signal a dismal prognosis, with median OS ranging from 10 to 13 months 28. Consequently, understanding the prognosis of patients with DM is crucial, and there is an urgent need to optimize treatment strategies for this cohort.

Platinum-based doublet chemotherapy, supplemented with either cetuximab 7, 8 or pembrolizumab 11, has long been the standard first-line treatment for metastatic laryngeal and hypopharyngeal cancers. In recent years, immunotherapies, particularly programmed cell death 1 (PD-1) inhibitors, have emerged as a promising area of research, showing the ability to improve OS in patients with PD-1 ligand 1 (PD-L1)-positive recurrent or metastatic head and neck squamous cell carcinoma (HNSCC) 11. However, the role of primary tumor surgery in the treatment of metastatic laryngeal and hypopharyngeal cancer remains uncertain. Notably, although the overall 5-year survival rates for most cancer types improved between 1975 and 2018, the survival rates for laryngeal cancer declined from 2012 to 2018 29. This decline in survival, observed in parallel with an increase in non-surgical therapies 30, 31, has raised concerns that the trend toward surgical de-escalation may be contributing to adverse outcomes 32. These findings suggest that the potential survival benefits of primary tumor surgery in metastatic laryngeal and hypopharyngeal cancer may have been underappreciated. Our multivariate Cox regression analysis revealed that primary tumor surgery significantly improved both OS and DSS in patients with metastatic laryngeal and hypopharyngeal cancers, whereas surgery on regional lymph nodes or metastatic sites alone did not confer the same benefit. This contrasts with previous studies that highlighted the survival advantages of surgery on metastatic sites. For example, selected patients with HNC who underwent resection of lung or liver metastases experienced 5-year survival rates exceeding 20% 33. Furthermore, metastasis-directed therapies, including surgery or stereotactic body radiotherapy (SBRT) 34, have shown promising survival benefits for patients with oligometastatic disease 35. The distinction between metastatic site surgery in this study and previous research lies in the broader patient cohort, as prior studies focused solely on oligometastatic disease, while this cohort includes a more heterogeneous group of patients with DM.

To further substantiate the benefits of primary tumor surgery observed in the Cox analysis, PSM was employed to adjust for variations in demographic and clinicopathological characteristics across subtypes. The PSM-adjusted data revealed that the survival benefits of primary tumor surgery were largely confined to laryngeal cancer, not hypopharyngeal cancer. This discrepancy may be attributed to the notoriously poor prognosis of hypopharyngeal cancer among HNC subtypes, as the anatomy of the hypopharynx facilitates insidious tumor progression 36, 37. Additionally, primary tumor surgery appeared to benefit only patients under 70 years of age, likely due to the poorer physical condition of elderly patients, for whom the complications of surgery may outweigh its potential benefits. Surgical interventions for advanced cancer are more complex, involving longer operative times, increased risks of postoperative complications, and functional decline 38. Several mechanisms may explain the survival benefits of primary tumor resection, including the prevention of locoregional progression, significant reduction in tumor burden 39, disruption of local tumor cell seeding 40, attenuation of tumor-derived growth factors and cytokines 41, and modification of the immune microenvironment 42. Collectively, these factors contribute to enhanced systemic immunity and improved responses to chemotherapy and immunotherapy. In addition to survival benefits, primary tumor surgery can preserve laryngeal function and offer psychological support by alleviating symptoms of locoregional tumor progression, which disrupts fundamental functions like breathing and swallowing. Although not all patients will benefit, the findings suggest that in carefully selected individuals, primary tumor surgery should be integrated into a multimodal treatment approach for metastatic disease, particularly for those exhibiting radioresistance and locoregional symptoms, thereby informing clinical decision-making.

Radiotherapy is another recommended therapeutic option for metastatic patients 15. However, its application is less extensively studied than chemotherapy, leaving the optimal patient subgroups for radiotherapy unclear. Our PSM-adjusted data indicated that, in general, radiotherapy significantly improved both OS and DSS for patients with metastatic laryngeal/hypopharyngeal cancer. However, this survival benefit was limited to patients with primary laryngeal cancer and the SCC subtype, while non-SCC individuals did not appear to benefit from radiotherapy. Additionally, 81 cases (after excluding 66 patients with indeterminate non-SCC histology) were examined. Among these, neuroendocrine carcinoma (53 cases) emerged as the predominant non-squamous malignancy. Most subtypes, including typical carcinoid 43, large cell neuroendocrine carcinoma 44, and paragangliomas 45, show poor sensitivity to radiotherapy, with surgery being the primary treatment modality for these tumors 46-48. While small cell carcinoma (30 cases) is exceptionally radiosensitive and radiotherapy can effectively control the primary tumor 49, its aggressive nature and rapid progression render it ineffective in prolonging survival 50. Additionally, adenoid cystic carcinoma (7 cases) 51 and sarcomas (2 cases) 52 are typically resistant to traditional radiotherapy, contributing to ongoing debates regarding its efficacy. Current literature on HNC largely focuses on SCC, and the management of non-SCC histologies relies predominantly on extrapolation from limited case series 53. Although non-SCC cases represent a smaller proportion of the population, they warrant more attention due to their distinct biological behavior, clinical course, and prognosis. Further comprehensive analysis of large population datasets for each non-SCC subtype is needed to enhance our understanding of these rare malignancies and inform the development of tailored treatment strategies. Radiotherapy has shown effectiveness across all age groups, consistent with several retrospective single-center studies, which report that oncologic outcomes for older patients receiving radiotherapy alone are comparable to those of younger patients 54, 55.

Additionally, a retrospective study found no significant differences between age groups in terms of treatment-related deaths, toxicity, treatment interruptions, or completion rates 56. However, older patients are often excluded from large randomized controlled trials, and selection bias typically favors the enrollment of healthier individuals. As a result, chemotherapy, the first-line treatment, is more commonly applied to younger patients, while the role of chemotherapy in elderly patients with HNC remains underexplored. Chemotherapy, being a systemic therapy, is associated with significant toxicity, and many elderly patients, particularly those with comorbidities or poor general health, are unable to tolerate the full course. In such cases, radiotherapy, a locoregional therapy, may be a viable alternative for patients who cannot tolerate chemotherapy. Notably, radiotherapy in older patients has not been associated with increased toxicity or adverse outcomes, making it a strong consideration for this population 57.

Several prior studies have investigated the impact of primary tumor treatment using Cox regression analysis. Borson *et al.* 58 conducted a single-center retrospective study involving 40 patients with metastatic HNSCC who underwent definitive surgery or chemoradiation at the primary site. Their findings revealed that definitive local treatment of the primary tumor, rather than treatment of metastatic lesions, significantly improved survival outcomes. Similarly, Zumsteg *et al.* 59 analyzed 3,269 patients with metastatic HNSCC from the National Cancer Data Base (NCDB), applying both Cox regression and PSM analysis. Their results highlighted that high-intensity local treatments, such as curative-dose radiotherapy or oncologic surgeries, substantially improved OS. Pan *et al.* 16 examined 446 patients with metastatic laryngeal cancer from the SEER database using Cox regression and a nomogram, suggesting that surgical treatment of the primary tumor improves survival. However, these studies lacked more detailed stratified analyses to quantify the survival benefit and a robust survival prediction model. Building on these earlier findings, this study focused on the survival of patients with laryngeal/hypopharyngeal cancer who developed DM. Comprehensive stratified survival analyses were performed based on the presence or absence of primary tumor surgery and radiotherapy. Notably, this study is the first to construct a machine learning model incorporating demographic and clinicopathological profiles of patients. The RSF model offers distinct advantages, as it is not constrained by assumptions like proportional hazards or log-linear relationships. Leveraging the strengths of random forests, including random search to prevent algorithmic overfitting 24, 25, the RSF model is well-suited for survival analysis, variable selection in high-dimensional data, and the analysis of competing risks. With satisfactory accuracy, our RSF model identified chemotherapy, primary tumor surgery, and radiotherapy as the top three independent prognostic factors for survival in patients with metastatic laryngeal/hypopharyngeal cancer. It also demonstrated the capability to predict patient survival outcomes. Optimizing patient outcomes remains a challenging task.

Despite the current challenges in survival rates, maintaining an optimistic therapeutic outlook is crucial. In some cases, integrating locoregional therapy with systemic treatment may offer a better prognosis. The complex decision-making process regarding tailored treatments for patients with metastatic laryngeal and hypopharyngeal cancer is influenced by patient preferences, tumor extent, clinician expertise, patient physical condition, and comorbidities.

Despite the promising findings, several limitations should be acknowledged. First, although the SEER database is comprehensive, its representation may not be entirely universal, particularly regarding racial diversity. Second, while the machine learning prognostic model demonstrated satisfactory accuracy, its reliability would benefit from further external validation. Third, while immunotherapy is increasingly recognized as an effective treatment modality in HNC, its impact on OS was not explored in this study due to the lack of immunotherapy data in the SEER database. Additionally, the SEER database does not provide detailed information on the specifics of radiotherapy, including the treatment field, fractionation, and dose. Fourth, this study aimed to explore the overall survival benefits of primary tumor surgery in patients with DM and to identify which subgroups could derive benefit. However, the lack of detailed surgical information for some patients and the variability in surgical procedures over the two-decade study period (with some now obsolete) prevented a more granular classification of surgical methods. Addressing these gaps will be a priority for future research, with plans to gather more detailed data, compare the effects of different treatments, and refine individualized therapeutic strategies. Fifth, the SEER database does not include information on oligometastatic disease, which may influence treatment decisions, as patients with limited metastatic burden are more likely to be considered candidates for surgery.

## Supplementary Material

Supplementary tables.

## Figures and Tables

**Table 1:** Univariate Cox analysis of variables achieved from the SEER database

	Univariate Cox analysis					
	OS			DSS		
	HR	95%CI	*P* Value	HR	95%CI	*P* Value
**Age at diagnosis**						
<70	reference			reference		
≥70	1.482	1.324-1.658	***	1.583	1.346-1.862	***
**Sex**						
female	reference			reference		
male	1.074	0.938-1.228	0.301	1.070	0.878-1.303	0.505
**Race**						
white	reference			reference		
black	1.048	0.922-1.191	0.475	0.873	0.714-1.066	0.182
others	0.902	0.710-1.145	0.395	1.067	0.773-1.472	0.694
**Histological type**						
Squamous	reference			reference		
non-Squamous	1.318	1.100-1.579	**	1.148	0.867-1.52	0.337
**Marital status**						
married	reference			reference		
single	1.159	1.019-1.318	*	1.060	0.8778-1.280	0.545
widow/divorced/others	1.280	1.132-1.448	***	1.171	0.979-1.402	0.085
**T stage**						
T1	reference			reference		
T2	1.105	0.888-1.375	0.371	0.950	0.700-1.290	0.743
T3	1.190	0.958-1.478	0.116	1.064	0.787-1.438	0.687
T4	1.118	0.911-1.373	0.284	0.955	0.718-1.269	0.751
**N stage**						
N0	reference			reference		
N1	1.060	0.882-1.275	0.534	0.937	0.714-1.229	0.638
N2	1.191	1.019-1.392	*	1.128	0.901-1.413	0.295
N3	1.346	1.087-1.667	**	1.239	0.907-1.692	0.178
**Primary site**						
glottis	reference			reference		
supraglottis	1.284	1.077-1.530	**	1.322	1.035-1.689	*
subglottis	0.872	0.568-1.340	0.533	0.728	0.377-1.403	0.343
larynx-others	1.508	1.233-1.844	***	1.482	1.117-1.965	**
hypopharynx	1.429	1.194-1.710	***	0.928	0.710-1.212	0.582
**Grade**						
well differentiated, Ⅰ	reference			reference		
moderately differentiated, Ⅱ	1.328	0.994-1.776	0.055	1.035	0.706-1.519	0.859
poorly differentiated, Ⅲ/Ⅳ	1.525	1.140-2.040	**	1.345	0.918-1.969	0.128
**Median household income** **(Inflation-adjusted)**						
<50,000$	reference			reference		
50,000-59,999$	0.958	0.802-1.144	0.635	0.928	0.705-1.221	0.592
60,000-69,999$	0.945	0.806-1.108	0.485	1.048	0.826-1.328	0.701
≥70,000$	0.981	0.851-1.130	0.785	1.113	0.901-1.376	0.321
**Chemotherapy**						
no/unknown	reference			reference		
yes	0.481	0.433-0.535	***	0.509	0.436-0.595	***
**Radiotherapy**						
no	reference			reference		
yes	0.572	0.516-0.635	***	0.582	0.499-0.678	***
**Primary tumor surgery**						
no	reference			reference		
yes	0.537	0.453-0.636	***	0.683	0.543-0.860	**
**Surgery on region lymph nodes**						
no	reference			reference		
yes	0.806	0.711-0.913	***	0.915	0.766-1.093	0.330
**Surgery on distant site**						
no	reference			reference		
yes	0.726	0.558-0.945	*	0.704	0.475-1.042	0.079
**Bone metastasis**						
no	reference			reference		
yes	1.409	1.206-1.647	***	1.539	1.228-1.93	***
**Liver metastasis**						
no	reference			reference		
yes	1.254	1.053-1.495	*	1.471	1.148-1.883	**
**Lung metastasis**						
no	reference			reference		
yes	1.206	1.054-1.379	**	1.048	0.861-1.275	0.642
**Brain metastasis**						
no	reference			reference		
yes	1.761	1.089-2.848	*	0.656	0.211-2.045	0.467
**Distant lymph nodes metastasis**						
no	reference			reference		
yes	1.025	0.811-1.296	0.836	1.163	0.837-1.614	0.368
**Other distant site metastasis**						
no	reference			reference		
yes	1.146	0.843-1.559	0.385	1.093	0.698-1.71	0.698

* *P* < 0.05, ** *P* < 0.01, *** *P* < 0.001 SEER: the Surveillance, Epidemiology, and End Results; OS: overall survival; DSS: disease-specific survival; HR: hazard ratio; CI: confidence interval.

**Table 2:** Multivariate Cox analysis of variables achieved from the SEER database

	Multivariate Cox analysis					
	OS			DSS		
	HR	95%CI	*P* Value	HR	95%CI	*P* Value
**Age at diagnosis**						
<70	reference			reference		
≥70	1.192	0.970-1.464	0.094	1.180	0.951-1.464	0.1319
**Sex**						
female	reference			reference		
male	/	/	/	/	/	/
**Race**						
white	reference			reference		
black	/	/	/	/	/	/
others	/	/	/	/	/	/
**Histological type**						
squamous	reference			reference		
non-squamous	1.091	0.650-1.834	0.741	/	/	/
**Marital status**						
married	reference			reference		
single	1.374	1.093-1.728	**	/	/	/
widow/divorced/others	1.039	0.839-1.286	0.727	/	/	/
**T stage**						
T1	reference			reference		
T2	/	/	/	/	/	/
T3	/	/	/	/	/	/
T4	/	/	/	/	/	/
**N stage**						
N0	reference			reference		
N1	1.277	0.926-1.761	0.135	/	/	/
N2	1.558	1.183-2.051	**	/	/	/
N3	1.834	1.256-2.679	**	/	/	/
**Primary site**						
glottis	reference			reference		
supraglottis	1.188	0.889-1.588	0.244	1.443	1.044-1.994	*
subglottis	0.322	0.126-0.824	*	0.848	0.362-1.987	0.7039
larynx-others	1.609	1.126-2.300	**	1.560	1.080-2.254	*
hypopharynx	1.266	0.926-1.730	0.140	0.941	0.662-1.338	0.7359
**Grade**						
well differentiated, Ⅰ	reference			reference		
moderately differentiated, Ⅱ	2.637	1.683-4.134	***	/	/	/
poorly differentiated, Ⅲ/Ⅳ	2.627	1.670-4.135	***	/	/	/
**Median household income** **(Inflation-adjusted)**						
<50,000$	reference			reference		
50,000-59,999$	/	/	/	/	/	/
60,000-69,999$	/	/	/	/	/	/
≥70,000$	/	/	/	/	/	/
**Chemotherapy**						
no/unknown	reference			reference		
yes	0.333	0.271-0.410	***	0.477	0.384-0.592	***
**Radiotherapy**						
no	reference			reference		
yes	0.512	0.424-0.620	***	0.668	0.545-0.819	***
**Primary tumor surgery**						
no	reference			reference		
yes	0.507	0.366-0.703	***	0.674	0.494-0.921	*
**Surgery on region lymph nodes**						
no	reference			reference		
yes	1.038	0.805-1.338	0.777	/	/	/
**Surgery on distant site**						
no	reference			reference		
yes	1.259	0.836-1.897	0.270	/	/	/
**Bone metastasis**						
no	reference			reference		
yes	1.015	0.802-1.286	0.901	1.478	1.172-1.864	***
**Liver metastasis**						
no	reference			reference		
yes	1.027	0.783-1.348	0.848	1.380	1.071-1.779	*
**Lung metastasis**						
no	reference			reference		
yes	0.975	0.800-1.188	0.799	/	/	/
**Brain metastasis**						
no	reference			reference		
yes	1.808	0.913-3.581	0.089	/	/	/
**Distant lymph nodes metastasis**						
no	reference			reference		
yes	/	/	/	/	/	/
**Other distant site metastasis**						
no	reference			reference		
yes	/	/	/	/	/	/

* *P* < 0.05, ** *P* < 0.01, *** *P* < 0.001 SEER: the Surveillance, Epidemiology, and End Results; OS: overall survival; DSS: disease-specific survival; HR: hazard ratio; CI: confidence interval.
