# Supplementary material for: Random survival forest predicts survival in patients with metastatic laryngeal and hypopharyngeal cancer and the prognostic benefits of surgery and radiotherapy
Source: J Cancer. 2025 Jan 1;16(2):603–21. doi: 10.7150/jca.103793 (PMC11685675; doi:10.7150/jca.103793)
Supplement: Supplementary file 1 — Supplementary tables. [file jcav16p0603s1.pdf]

Supplementary Table 1： Baseline characteristics in patients with metastatic laryngeal or hypopharyngeal cancer.

**Characteristic**		**Cases**	**%**
**Age at diagnosis**	<70	1132	69.62%
	≥70	494	30.38%
**Sex**	female	295	18.14%
	male	1331	81.86%
**Histological type**	squamous	1479	90.96%
	non-squamous	147	9.04%
**Primary site**	glottis	197	12.12%
	supraglottis	620	38.13%
	subglottis	31	1.91%
	larynx-others	260	15.99%
	hypopharynx	518	31.86%
**Marital status**	married	608	37.39%
	single	471	28.97%
	widow/divorced/others	547	33.64%
**Race**	white	1205	74.11%
	black	331	20.36%
	others	87	5.35%
	unknown	3	0.19%
**T stage**	T1	135	8.30%
	T2	325	19.99%
	T3	335	20.60%
	T4	599	36.84%
	unknown	232	14.27%
**N stage**	N0	243	14.95%
	N1	275	16.91%
	N2	781	48.03%
	N3	161	9.90%
	unknown	166	10.21%
**Grade**	well differentiated; I	65	4.00%
	moderately differentiated; II	520	31.98%
	poorly differentiated; III/IV	495	30.44%

	unknown	546	33.58%
Median household income (Inflation-adjusted)	<49,999$	317	19.50%
	50,000-59,999$	251	15.44%
	60,000-69,999$	366	22.51%
	≥70,000$	692	42.56%
Chemotherapy	no/unknown	687	42.25%
	yes	939	57.75%
Radiotherapy	no	774	47.60%
	yes	852	52.40%
Primary tumor surgery	no	1437	88.38%
	yes	189	11.62%
Surgery on region lymph nodes	no	1245	76.57%
	yes	368	22.63%
	unknown	13	0.80%
Surgery on distant site	no	1555	95.63%
	yes	69	4.24%
	unknown	2	0.12%
Bone metastases	no	818	50.31%
	yes	242	14.88%
	unknown	566	34.81%
Liver metastases	no	892	54.86%
	yes	170	10.46%
	unknown	564	34.67%
Lung metastases	no	438	26.94%
	yes	622	38.25%
	unknown	566	34.81%
Brain metastasis	no	1036	63.72%
	yes	18	1.11%
	unknown	572	35.18%
Distant lymph nodes metastases	no	358	22.02%
	yes	128	7.87%
	unknown	1140	70.11%
Distant other metastases	no	419	25.77%

	yes	66	4.06%
	unknown	1141	70.17%

Supplementary Table 2. Comparison of patient characteristics according to the performance of primary tumor surgery or not before and after propensity score matching (PSM)

Characteristics	Unmatched Cohort					1:2 propensity score matched (PSM) Cohort				
	Surgery		Surgery not given		Unadjusted	Surgery		Surgery not given		PSM-adjusted
	N=186	%	N=1317	%	*P* value	N=132	%	N=226	%	*P* value
**Age at diagnosis**					＜0.001					0.794
<70	157	84.41%	905	68.72%		108	81.82%	181	80.09%	
≥70	29	15.59%	412	31.28%		24	18.18%	45	19.91%	
**Histological type**					0.390					1.000
squamous	167	89.78%	1211	91.95%		119	90.15%	204	90.27%	
non-squamous	19	10.22%	106	8.05%		13	9.85%	22	9.73%	
**Marital status**					0.060					0.187
married	82	44.09%	484	36.75%		52	39.39%	90	39.82%	
single	55	29.57%	378	28.70%		38	28.79%	82	36.28%	
widow/divorced/others	49	26.34%	455	34.55%		42	31.82%	54	23.89%	
**N stage**					0.050					0.846
N0	41	22.04%	180	13.67%		23	17.42%	37	16.37%	
N1	32	17.20%	227	17.24%		26	19.70%	46	20.35%	
N2	80	43.01%	648	49.20%		58	43.94%	105	46.46%	
N3	18	9.68%	137	10.40%		12	9.09%	23	10.18%	
unknown	15	8.06%	125	9.49%		13	9.85%	15	6.64%	
**Primary site**					＜0.001					0.852
glottis	43	23.12%	140	10.63%		25	18.94%	39	17.26%	
supraglottis	73	39.25%	507	38.50%		52	39.39%	96	42.48%	
subglottis	6	3.23%	22	1.67%		2	1.52%	2	0.88%	
larynx-others	31	16.67%	203	15.41%		21	15.91%	29	12.83%	
hypopharynx	33	17.74%	445	33.79%		32	24.24%	60	26.55%	
**Grade**					＜0.001					0.908
well differentiated; I	16	8.60%	46	3.49%		7	5.30%	11	4.87%	
moderately differentiated; II	65	34.95%	430	32.65%		46	34.85%	71	31.42%	
poorly differentiated; III/IV	80	43.01%	381	28.93%		55	41.67%	99	43.81%	
unknown	25	13.44%	460	34.93%		24	18.18%	45	19.91%	
**Chemotherapy**					0.118					0.484
no/unknown	82	44.09%	498	37.81%		53	40.15%	81	35.84%	
yes	104	55.91%	819	62.19%		79	59.85%	145	64.16%	
**Radiotherapy**					0.562					0.747
no	78	41.94%	586	44.50%		58	43.94%	94	41.59%	
yes	108	58.06%	731	55.50%		74	56.06%	132	58.41%	
**Surgery on region lymph nodes**					＜0.001					0.290
no	66	35.48%	1074	81.55%		64	48.48%	125	55.31%	

yes	117	62.90%	233	17.69%		66	50.00%	100	44.25%	
unknown	3	1.61%	10	0.76%		2	1.52%	1	0.44%	
**Surgery on distant site**					＜0.001					0.852
no	159	85.48%	1276	96.89%		124	93.94%	213	94.25%	
yes	26	13.98%	41	3.11%		8	6.06%	13	5.75%	
unknown	1	0.54%	0	0.00%		0	0.00%	0	0.00%	
**Bone metastasis**					0.185					0.998
no	83	44.62%	674	51.18%		63	47.73%	107	47.35%	
yes	49	26.34%	191	14.50%		18	13.64%	31	13.72%	
unknown	55	29.57%	452	34.32%		51	38.64%	88	38.94%	
**Liver metastasis**					0.102					0.991
no	99	53.23%	727	55.20%		71	53.79%	121	53.54%	
yes	12	6.45%	139	10.55%		10	7.58%	18	7.96%	
unknown	75	40.32%	451	34.24%		51	38.64%	87	38.50%	
**Lung metastasis**					0.232					0.657
no	51	27.42%	351	26.65%		35	26.52%	51	22.57%	
yes	61	32.80%	512	38.88%		46	34.85%	87	38.50%	
unknown	74	39.78%	454	34.47%		51	38.64%	88	38.94%	
**Brain metastasis**					0.064					1.000
no	109	58.60%	847	64.31%		80	60.61%	138	61.06%	
yes	0	0.00%	17	1.29%		0	0.00%	0	0.00%	
unknown	77	41.40%	453	34.40%		52	39.39%	88	38.94%	

Supplementary Table 3. Median overall survival (month) of patients with and without primary tumor surgery

Characteristic	Primary Tumor Surgery				
	No		Yes		
	Median OS (month)	95% CI	Median OS (month)	95% CI	Log-rank *P*
**Overall**	9.0	7.0-10.0	13.0	10.0-16.0	***
**Age**					
<70	10.0	7.0-11.0	14.0	11.0-19.0	***
≥70	5.0	4.0-9.7	9.5	3.0-15.0	0.220
**Primary site**					
larygnx	8.0	6.0-11.0	15.0	10.0-21.0	***
hypopharynx	9.0	5.6-11.0	11.0	6.6-14.0	0.160
**N stage**					
N0	10.0	5.0-14.4	15.0	10.0-24.0	0.093
N1	8.5	5.0-13.0	19.5	9.5-38.0	**
N2	9.0	6.0-11.0	14.0	8.0-20.5	*
N3	10.0	3.0-16.9	5.5	3.8-11.4	0.220

* $P < 0.05$, ** $P < 0.01$, *** $P < 0.001$; OS: overall survival

Supplementary Table 4. Comparison of patient characteristics according to radiotherapy before and after propensity score matching (PSM)

Characteristics	Unmatched Cohort					1:1 propensity score matched (PSM) Cohort				
	Radiotherapy		Radiotherapy not given		Unadjusted	Radiotherapy		Radiotherapy not given		PSM-adjusted
	N=839	%	N=664	%	*P* value	N=541	%	N=541	%	*P* value
**Age at diagnosis**					0.013					0.789
<70	615	73.30%	447	67.32%		472	87.25%	475	87.80%	
≥70	224	26.70%	217	32.68%		69	12.75%	66	12.20%	
**Histological type**					＜0.001					0.823
squamous	791	94.28%	587	88.40%		499	92.24%	496	91.68%	
non-squamous	48	5.72%	77	11.60%		42	7.76%	45	8.32%	
**Marital status**					0.523					0.921
married	325	38.74%	241	36.30%		205	37.89%	200	36.97%	
single	233	27.77%	200	30.12%		159	29.39%	158	29.21%	
widow/divorced/others	281	33.49%	223	33.58%		177	32.72%	183	33.83%	
**N Stage**					0.001					0.943
N0	111	13.23%	110	16.57%		82	15.16%	90	16.64%	
N1	159	18.95%	100	15.06%		91	16.82%	88	16.27%	
N2	416	49.58%	312	46.99%		265	48.98%	267	49.35%	
N3	94	11.20%	61	9.19%		59	10.91%	53	9.80%	
unknown	59	7.03%	81	12.20%		44	8.13%	43	7.95%	
**Primary site**					0.005					0.649
glottis	102	12.16%	81	12.20%		73	13.49%	68	12.57%	
supraglottis	343	40.88%	237	35.69%		217	40.11%	202	37.34%	
subglottis	13	1.55%	15	2.26%		10	1.85%	8	1.48%	
larynx-others	106	12.63%	128	19.28%		73	13.49%	88	16.27%	
hypopharynx	275	32.78%	203	30.57%		168	31.05%	175	32.35%	
**Grade**					0.274					0.836
well differentiated; I	31	3.69%	31	4.67%		22	4.07%	24	4.44%	
moderately differentiated; II	293	34.92%	202	30.42%		168	31.05%	179	33.09%	
poorly differentiated; III/IV	252	30.04%	209	31.48%		172	31.79%	171	31.61%	
unknown	263	31.35%	222	33.43%		179	33.09%	167	30.87%	
**Chemotherapy**					＜0.001					0.537
no/unknown	237	28.25%	343	51.66%		218	40.30%	229	42.33%	
yes	602	71.75%	321	48.34%		323	59.70%	312	57.67%	
**Primary tumor surgery**					0.562					0.854
no	731	87.13%	586	88.25%		472	87.25%	475	87.80%	
yes	108	12.87%	78	11.75%		69	12.75%	66	12.20%	

**Surgery on region lymph nodes**					0.449					0.383
no	638	76.04%	502	75.60%		405	74.86%	412	76.16%	
yes	196	23.36%	154	23.19%		132	24.40%	128	23.66%	
unknown	5	0.60%	8	1.20%		4	0.74%	1	0.18%	
**Surgery on distant site**					0.542					0.774
no	798	95.11%	637	95.93%		514	95.01%	517	95.56%	
yes	40	4.77%	27	4.07%		27	4.99%	24	4.44%	
unknown	1	0.12%	0	0.00%		0	0.00%	0	0.00%	
**Bone metastasis**					0.036					0.975
no	411	48.99%	346	52.11%		275	50.83%	276	51.02%	
yes	111	13.23%	107	16.11%		275	50.83%	276	51.02%	
unknown	317	37.78%	211	31.78%		186	34.38%	183	33.83%	
**Liver metastasis**					＜0.001					0.787
no	465	55.42%	361	54.37%		304	56.19%	299	55.27%	
yes	61	7.27%	90	13.55%		54	9.98%	61	11.28%	
unknown	313	37.31%	213	32.08%		183	33.83%	181	33.46%	
**Lung metastasis**					0.001					0.792
no	232	27.65%	170	25.60%		153	28.28%	144	26.62%	
yes	286	34.09%	287	43.22%		204	37.71%	213	39.37%	
unknown	321	38.26%	207	31.17%		184	34.01%	184	34.01%	
**Brain metastasis**					0.046					0.94
no	511	60.91%	445	67.02%		351	64.88%	352	65.06%	
yes	11	1.31%	6	0.90%		4	0.74%	5	0.92%	
unknown	317	37.78%	213	32.08%		186	34.38%	184	34.01%	

Supplementary Table 5. Median overall survival (in months) of patients with and without radiotherapy

Characteristic	Radiotherapy				
	No		Yes		
	Median OS (month)	95% CI	Median OS (month)	95% CI	Log-rank *P*
**Overall**	6.0	5.0-7.0	9.0	8.0-10.0	***
**Age**					
<70	7.0	6.0-8.0	10.0	8.0-11.0	***
≥70	4.0	3.0-5.0	8.0	7.0-9.0	***
**Primary site**					
larygnx	6.0	5.0-7.0	10.0	8.0-12.0	***
hypopharynx	6.0	5.0-8.0	8.0	6.9-9.0	0.057
**N stage**					
N0	5.5	4.0-8.0	13.5	10.5-16.5	***
N1	6.0	5.0-8.5	10.0	7.0-14.0	**
N2	6.0	5.0-7.0	8.0	7.0-9.0	***
N3	5.0	3.0-8.0	8.0	6.0-10.0	0.066
**Histological type**					
squamous	7.0	6.0-8.0	15.0	12.0-18.0	***
non-squamous	10.4	7.4-14.2	8.0	6.0-11.7	0.240

* $P < 0.05$, ** $P < 0.01$, *** $P < 0.001$；OS：overall survival
